# The Main Task of Urban Public Health: Narrowing the Health Gap Between the Poor and the Rich

**DOI:** 10.3389/ijph.2022.1605084

**Published:** 2022-09-28

**Authors:** Benedikt Wicki

**Affiliations:** ^1^ Swiss Tropical and Public Health Institute (Swiss TPH), Basel, Switzerland; ^2^ University of Basel, Basel, Switzerland

**Keywords:** health inequalites, policy, environmental health, urban public health, environmental inequalities, gentrification

**The IJPH series “Young Researcher Editorial” is a training project of the Swiss School of Public Health.**

Worldwide, urban centers keep growing: an estimated 68% of the world’s population will live in cities by 2050 [1]. Typical urban environmental stressors can harm the health of city dwellers, who may suffer from air pollution, transportation noise, and urban heat islands [2]. Maintaining urban access to green space decreases stress levels and promotes physical and mental health [3], but urban stressors and mitigators are unequally distributed. In Europe, communities of lower socioeconomic status (SES) are often exposed to higher levels of air pollution, transportation noise, and heat [4], at the same time that low SES is associated with increased risk of cardiovascular and pulmonary diseases, metabolic disorders, and lower life-expectancy [5]. As the evidence conclusively demonstrates, low socioeconomic status is associated with worse health, but the risks can be mitigated by providing resources that actively improve health and help people cope with stressors.

Since people of lower SES are both more often exposed and more vulnerable to environmental stressors, increasing attention has been devoted to reducing their risk factors in urban areas. The field of urban public health in general, which integrates health perspectives into urban planning, is growing [6]. There is evidence that urban planning and transportation interventions can change an urban population’s behavior, reduce their exposure to environmental hazards, and improve their social environment. Developing and maintaining human-friendly urban spaces can improve population health. Typical urban public health interventions include changing the way land is used, e.g., creating green spaces, reducing traffic volume and speed, promoting activity, and increasing access to public transportation [7].

Unfortunately, the potential benefits of these strategies may make an urban area less accessible to the population they were designed to serve. Improving a low SES neighborhood in a free market economy can also drive up rents if market mechanisms are allowed to run unchecked. Making an area more appealing can drive gentrification, ultimately pricing out the original inhabitants. Gentrification is common when poorer urban areas are upgraded. When the lower SES population moves out, the effect of urban public health interventions is lost and health inequality may actually increase. To avoid this and ensure urban public health approaches have maximum beneficial effect, politicians and urban planners should adopt the following three strategies.

First, urban improvement actions should target the areas where their impact will be largest: areas with low SES populations and high levels of exposure to environmental stressors. Health impact assessment to identify such areas and regular monitoring of urban public health indicators should be integrated into city planning [8].

Second, public health professionals, policy makers, and city planners should work together to create appropriate legal, administrative and technical frameworks that account for local conditions and the specific needs of the local community. Engaging inhabitants in urban planning will encourage them to co-create their future living environment, e.g., in the form of urban living labs.

Third, actions should target populations and not just geographies. Since persons of low SES will continue to be more vulnerable to environmental stressors, planners should aim to improve their living conditions because this will provide the greatest public health benefits. These strategies must be implemented carefully, to ensure that the low SES inhabitants of an area can afford to keep living there. Coupling interventions to rent controls could prevent gentrification, as could other policy solutions mentioned in the Council of Europe’s 2020 policy brief, “Managing Gentrification” [9].

The urban health indicators that planners progressively monitor should focus on population groups, and not just on between-city or area comparisons. For example, monitoring the effects of interventions on area demographics could allow planners to identify early signs of gentrification, so a city can take appropriate counter measures. If policy makers are aware that environmental improvements potentially lead to gentrification and take steps to prevent it, they will be able to decrease health inequality.

Urban public health advocates should devise policies that narrow the health gap between poor and wealthy socioeconomic groups. Scientists can do their part to support this process, through targeted research that generates high-quality evidence on health inequality. The better we understand the reasons behind and the effects of environmental inequality, the intersecting factors and underlying causes, the better we will be able to plan our urban environments to serve lower SES populations. Applied appropriately, careful urban planning can play a key role in reducing health inequality.
